# Heterotic pools in African and Asian origin populations of pearl millet [*Pennisetum glaucum* (L.) R. Br.]

**DOI:** 10.1038/s41598-021-91568-7

**Published:** 2021-06-09

**Authors:** K. Sudarshan Patil, K. D. Mungra, Shashibhushan Danam, Anil Kumar Vemula, Roma R. Das, Abhishek Rathore, S. K. Gupta

**Affiliations:** 1 International Crops Research Institute for the Semi-Arid Tropics (ICRISAT), Hyderabad, Telangana India; 2 Professor Jayashankar Telangana State Agricultural University (PJTSAU), Hyderabad, Telangana India; 3 Junagadh Agricultural University (JAU), Jamnagar, Gujarat India

**Keywords:** Plant breeding, Plant breeding

## Abstract

Forty-five African or Asian origin pearl millet populations bred either in Africa or Asia were investigated to generate information on heterotic pools. They were clustered into seven groups (G1 to G7) when genotyped, using 29 highly polymorphic SSRs. Fourteen parental populations representing these seven marker-based groups were crossed in diallel mating design to generate 91 population hybrids. The hybrids evaluated at three locations in India showed mean panmictic mid-parent heterosis (PMPH) and better-parent heterosis (PBPH) for grain yield ranging from − 21.7 to 62.08% and − 32.51 to 42.99%, respectively. Higher grain yield and heterosis were observed in G2 × G6 (2462 kg ha^−1^, 43.2%) and G2 × G5 (2455 kg ha^−1^, 42.8%) marker group crosses compared to the most popular Indian open-pollinated variety (OPV) ICTP 8203. Two heterotic groups, Pearl millet Population Heterotic Pool-1 (PMPHP-1) comprising G2 populations and Pearl millet Population Heterotic Pool-2 (PMPHP-2) comprising G5 and G6 populations, were identified based on hybrid performance, heterosis and combining ability among marker group crosses. Population hybrids from two heterotic groups, PMPHP-1 × PMPHP-2 demonstrated PMPH of 14.75% and PBPH of 6.8%. Populations of PMPHP-1 had linkages with either African or Asian origin populations, whereas PMPHP-2 composed of populations originating in Africa and later bred for Asian environments. Results indicated that parental populations from the two opposite heterotic groups can be used as base populations to derive superior inbred lines to develop high yielding hybrids/cultivars.

## Introduction

Pearl millet [*Pennisetum glaucum* (L.) R. Br.] is the sixth most important cereal food crop after wheat, rice, maize, barley and sorghum in terms of global area (~ 31 million ha) and production (~ 28 Mt), with a majority of the area being cultivated in Africa (~ 18 million ha) and Asia (~ 10 million ha)^1^. India is the largest producer of pearl millet in the world with 9.70 Mt produced from 7.5 m ha^2^. Pearl millet’s cultivation has lately expanded to non-traditional areas like Brazil^3^, USA, Canada, Mexico, West Asia and North Africa (WANA) and Central Asia^4^. Globally, it serves as a staple food source to more than 90 million people and is also an important source of fodder for animals in the arid and semi-arid regions of sub-Saharan Africa (SSA) and South Asia (SA). Pearl millet is a climate resilient crop and important fodder crop for livestock due to its higher photosynthetic efficiency (C_4_ plant) and dryer matter production capacity compared to most other cereals. Hence, it can adapt better in adverse agro-climatic regions in comparison to other cereal crops and produce economic yields in marginal ecologies^5^.

Pearl millet offers great opportunities to exploit heterosis through hybrid development due to its high rate of cross-pollination (> 85% outcrossing) arising from its protogynous nature, huge genetic variability as well as the availability of stable cytoplasmic genetic male sterility (CGMS) system. India’s pearl millet breeding program continues to exploit heterosis, and has attained current grain yields of 1305 kg ha^−1^ compared to 305 kg ha^−1^ in the 1950s through the cultivation of single-cross hybrids^2^. On the contrary, pearl millet productivity in Africa has remained stagnant over the last three to four decades, though the region has seen a 5% increase per annum in area^6^. This has been due to the cultivation of conventional landraces and a few improved Open Pollinated Varieties (OPVs)^7^. It is imperative to diversify and broaden the genetic base of pearl millet germplasm in order to maintain the current rate of productivity of hybrids in Asian countries like India, and also to enhance productivity in SSA to either support their population improvement program or to initiate hybrid breeding.

Diversity studies based on agro-morphological traits in pearl millet have demonstrated huge variability among African germplasms^8–10^ and Asian germplasms^11,12^. Studies based on different molecular markers have reported significant genetic variation among pearl millet germplasms^13–18^. In addition to diversity, pearl millet population/landrace-based hybrids have shown significant heterosis among Asian populations^19–21^ and African germplasms^22–25^.

Over the past 45 years, breeders at the International Crops Research Institute for the Semi-Arid Tropics (ICRISAT) have developed a diverse range of gene pools, populations, trait-based composites and OPVs using germplasm originating in Africa and/or Asia^4,26^. These ICRISAT-developed populations have shown significant heterosis for grain yield and linked traits^27–29^. This evidence of higher heterosis using diverse pearl millet germplasms suggested the identification of heterotic groups to enhance current levels of genetic gain. Extensive studies have been done to explore the possibility of formulating heterotic groups in maize^30–32^, rye^33^, sunflower^34^, sorghum^35^, triticale^36^ and rice^37,38^ using hybrid parental lines/inbred lines. Recently, heterotic groups have been identified in pearl millet hybrid parental lines by Ramya et al.^39^, Singh and Gupta^40^ and Gupta et al.^41^. However, information on landrace/population-based heterotic grouping is limited in most of the crops. In maize, Reif et al.^42,43^ formed heterotic pools in populations based on the relationship between simple sequence repeat (SSR) diversity, combining ability and heterosis, while Gurung et al.^44^ and Laude and Carena^45^ identified heterotic pools based on combining ability patterns and morphological diversity. A recent study on West African pearl millet populations failed to identify heterotic groups and found high levels of genetic admixture in the germplasm that could be the reason^24^. A more recent study identified complimentary heterotic patterns between OPVs of Senegal and Niger origin based on the combining ability pattern^25^. These two studies used a limited number of populations belonging to only the West African region and identified superior combining ability patterns for the West African breeding program. We recently reported that significant genetic diversity exists among African and Asian origin/bred pearl millet populations^46^. The same set of African and Asian origin/bred pearl millet populations were further investigated to generate information on patterns of heterotic pools, whose results are being reported here.

## Results

### Genetic distance (GD) and grouping of pearl millet populations

**Figure 1:**
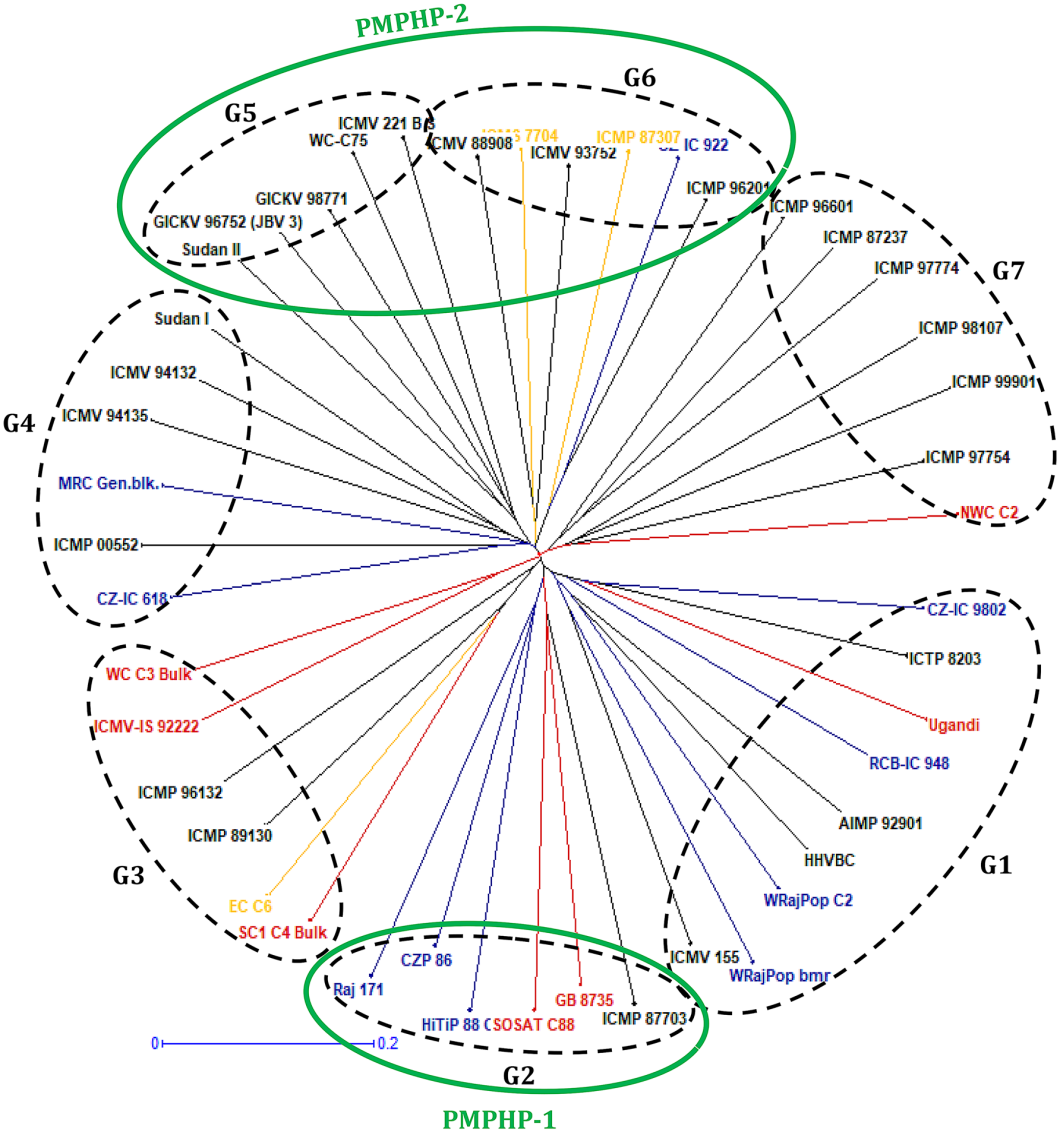
Unweighted Neighbor-joining tree of 45 pearl millet populations [10- As-As (Asian origin and Asian bred), 7 Af-Af (African origin and African bred), 3 (As × Af)-As {(Asian × African) origin and Asian bred} and 25 Af-As (African origin and Asian bred populations)] based on Modified Rogers distance (MRD) matrix using SSR markers. The blue color denotes As-As populations, the red color Af-Af, the yellow color (As × Af)-As and the black color Af-As populations. The G2 group represents PMPHP-1 (Pearl millet population heterotic pool-1) and G5 + G6 represents PMPHP-2 (Pearl millet population heterotic pool-2).

A total of 435 alleles were found to the 29 SSR markers with an average of 15 alleles per locus (Table 1) in 45 pearl millet populations. Higher mean values were observed for gene diversity (H_e_) (0.75), observed heterozygosity (H_o_) (0.31) and polymorphism information content (PIC) (0.72) (Table 1). Parental populations were found distributed across seven clusters (mentioned as marker groups and designated G1 to G7). Marker groups G4, G5, G6 and G7 were dominated by a majority of Af-As (African origin-Asian bred) populations, while Af-Af (African origin-African bred) and As-As (Asian origin-Asian bred) populations were distributed across all the seven groups (Fig. 1). Genetic distance between the 21 marker group crosses varied from 0.67 (G2 × G5) to 0.85 (G3 × G5). Results of Analysis of molecular variance (AMOVA) for 45 populations showed highly significant genetic variation within the individuals of the population (88.94%) compared to the genetic variation found between populations (11.06%)^46^. AMOVA for the seven marker groups also showed significantly higher genetic variation among individuals within populations (51.7%) and variation within individuals (46.4%) compared to the significant genetic variation found among the marker groups (1.9%) (Table 2). Population differentiation (F*st*) values in the AMOVA were found significant between seven marker groups, indicating they are significantly different from each other (Table 3).

**Table 1:** Range and mean values for allelic composition, gene diversity, heterozygosity and polymorphism information content (PIC) of 29 simple sequence repeat (SSR) loci in 45 pearl millet populations.

Diversity parameters	Minimum	Maximum	Mean
Number of alleles	6	32	15
Major allele frequency	0.07	0.61	0.37
Gene diversity (H_e_)	0.57	0.96	0.75
Heterozygosity (H_o_)	0.10	0.67	0.31
Polymorphism information content	0.51	0.95	0.72

**Table 2:** Analyses of molecular variance (AMOVA) and fixation indices for the seven marker groups.

Source of variation	df	Sum of squares	Variance components	Percentage of variation
Among marker groups	6	217.3	0.13 Va	1.9
**Among individuals**				
Within populations	713	7351.3	3.56 Vb	51.7
Within individuals	720	2299.0	3.19 Vc	46.4
Total	1439	9867.6	6.88	

Fixation indices				
F*is*	0.53***			
F*st*	0.02***			
F*it*	0.54***

**Table 3:** Pairwise F*st* matrix for the seven marker groups.

Marker group	1	2	3	4	5	6	7
1	0						
2	0.02654**	0					
3	0.02183**	0.02211**	0				
4	0.01685**	0.03190**	0.01685**	0			
5	0.02497**	0.03602**	0.01503**	0.01610**	0		
6	0.02676**	0.03705**	0.01552**	0.01621**	0.00796**	0	
7	0.02202**	0.03448**	0.00972**	0.01392**	0.01013**	0.00978**	0

*, **, ***Significant at 0.05, 0.01, < 0.001 levels of probability, respectively.

### Combining ability variance, performance of parental populations per se, hybrid performance and combining ability effects

The general combining ability (GCA) and specific combining ability (SCA) variances and their interactions with the environment were found significant, except for GCA × environment interaction. The σ^2^_*GCA*_/σ^2^_*SCA*_ ratio was 0.39 for grain yield (Table 4). Table 5 shows details of the per se performance of parental populations and population hybrids and GCA and SCA effects for grain yield across three environments. Grain yield of parental populations varied from 1322 kg ha^−1^ (ICMS 7704) to 2667 kg ha^−1^ (Sudan I) with an overall mean of 2133 kg ha^−1^, while that of population hybrids varied from 1652 kg ha^−1^ (EC C6 × GB 8735) to 2992 kg ha^−1^ (GB 8735 × ICMP 87307) with an overall mean of 2247 kg ha^−1^. The GCA effects for grain yield per hectare varied from − 233.7 (*P* < 0.01) (EC C6) to 130.0 (*P* < 0.01) (Sudan I) among parental populations. Of the 14 parental populations, 5 parents exhibited significantly positive GCA effects, whereas 4 showed significantly negative GCA effects. Among population hybrids, SCA effects varied from − 405.6 (*P* < 0.01) (EC C6 × GB 8735) to 633.2 (*P* < 0.01) (GB 8735 × ICMP 87307). Twenty-four hybrids had significant SCA effects; of these, 12 had positive and 12 had negative SCA effects. The mean grain yield of 91 population hybrids (2247 kg ha^−1^) was much higher than that of parental populations (2133 kg ha^−1^). Of the 91 population hybrids, 48 had positive SCA, and of these 12 had significant positive SCA. Hybrid GB 8735 × ICMP 87307 showed highest significant SCA effect while having H^+^ gca × H^+^ gca parental combination, followed by EC C6 × HHVBC with H^−^ gca × L^+^ gca combination. Fourteen population hybrids had H^−^ gca × H^+^ gca parental combination (Table 5). High positive significant correlation (r = 0.70, *P* < 0.01) was found between GCA for grain yield and mean grain yield per se of the parental populations.

**Table 4:** Variance components of General Combining Ability (GCA) and Specific Combining Ability (SCA) from diallel analysis method-II for grain yield across three locations.

Source of variation	df	Variance component	*P*-value
${\sigma }^{2}GCA$	13	7521.4	0.0642
${\sigma }^{2}SCA$	90	19,224.0	0.0041
${\sigma }^{2}GCA$ × Environment	13	3325.4	0.1134
${\sigma }^{2}SCA$ × Environment	90	38,692.0	< .0001
${\sigma }^{2}GCA$ /${\sigma }^{2}SCA$	–	0.39	
Predictability ratio	–	0.44

**Table 5:** Combining ability effects (SCA-below diagonal and GCA-bottom row), parental populations’ grain yield (in bold and on diagonal in kg ha^−1^) and population hybrids’ grain yield (above diagonal in kg ha^−1^) across three locations.

Population	EC C6	ICMP 98107	NWC C2	ICMP 87237	GB 8735	GICKV 98771	ICMS 7704	ICMV 155	SOSAT C88	Sudan I	Ugandi	MRC GB	HHVBC	ICMP 87307
EC C6	**1792**	1671	1755	2081	1652	1967	1937	2242	2382	2150	2024	1878	2508	1906
ICMP 98107	− 333.67**	**2475**	2051	2409	1950	2110	2197	2457	2286	2561	2029	2594	2042	2315
NWC C2	− 116.07	− 83.72	**2017**	2581	2041	2339	2191	1985	1971	2093	2243	2006	2112	2236
ICMP 87237	− 18.70	58.55	360.03**	**2206**	2540	2411	2370	2424	2579	2226	2428	1932	2270	2460
GB 8735	− 405.76**	− 338.59**	− 99.82	138.25	**2278**	2440	2139	2210	2596	2462	2362	1964	2482	2992
GICKV 98771	− 61.01	− 162.65	181.36	38.11	111.69	**2193**	2280	2273	2469	2374	2378	2309	2304	2023
ICMS 7704	46.17	59.63	152.58	145.15	− 41.17	96.17	**1322**	2472	2338	2257	2298	2205	2269	2318
ICMV 155	266.43*	233.87*	− 103.99	131.37	− 57.43	25.51	352.29**	**1729**	2274	2532	2007	2486	2283	1980
SOSAT C88	287.68**	− 52.71	− 234.59*	147.44	215.13*	118.06	115.40	− 19.55	**2320**	2194	2032	2444	2344	2377
Sudan I	16.43	191.97	− 173.34	− 252.52*	44.44	− 35.03	− 3.94	188.79	− 265.02*	**2667**	2626	2116	2228	2487
Ugandi	− 1.35	− 212.53	114.38	82.19	39.14	88.46	147.13	− 201.24	− 297.19**	241.20*	**2494**	2051	2190	2055
MRC GB	− 53.28	405.07**	− 35.03	− 344.58**	− 255.74*	117.61	150.79	367.98**	195.70	− 169.23	− 135.89	**1989**	2122	2233
HHVBC	478.21**	− 203.9	− 36.98	− 92.16	153.39	25.43	98.02	44.10	− 20.21	− 182.14	− 66.39	− 68.14	**2082**	2601
ICMP 87307	− 167.49	− 14.31	56.65	69.90	633.02**	− 313.43**	107.45	− 311.46**	− 11.07	55.34	− 258.56*	19.21	275.16*	**2304**
GCA	− 233.77**	**8.31**	− 111.68**	104.71**	55.77*	**38.09**	− 100.14**	− **24.52**	89.42**	130.00**	**16.26**	− 74.14**	**28.59**	73.11*

*, **Significant at 0.05 and 0.01 levels of probability, respectively.

In 21 marker-based hybrid groups, mean grain yield of the marker group varied from 1836 kg ha^−1^ (G3 × G7) to 2462 kg ha^−1^ (G2 × G6) (Table 6). Based on the performance of group crosses for grain yield, group cross G2 × G6 (2462 kg ha^−1^) had highest grain yield followed by G2 × G5 (2455 kg ha^−1^) and G4 × G5 (2342 kg ha^−1^).

**Table 6:** Genetic distance (GD), hybrid yield, yield heterosis (PMPH, PBPH and PCH) and specific combining ability (SCA) for 21 marker group crosses in pearl millet across three locations.

Marker group cross	Number of hybrids	Mean GD	HGY (kg ha^−1^)	PMPH (%)	PBPH (%)	PCH over ICTP 8203 (%)	PCH over ProAgro 9444 (%)	SCA
G2 × G6	4	0.73	2461.7	20.2	6.8	43.2	− 14.8	93.3
G2 × G5	2	0.67	2454.7	9.3	6.8	42.8	− 15.1	198.4
G4 × G5	2	0.72	2341.8	4.1	− 2.8	36.2	− 19.0	131.3
G1 × G4	6	0.74	2340.8	6.7	− 2.3	36.1	− 19.0	− 167.2
G1 × G5	3	0.71	2318.4	8.4	1.4	34.8	− 19.8	57.4
G4 × G6	4	0.71	2295.4	12.6	− 3.6	33.5	− 20.6	− 7.6
G6 × G7	6	0.73	2294.8	15.1	0.4	33.4	− 20.6	1.2
G5 × G7	3	0.83	2286.6	3.7	0.4	33.0	− 20.9	34.4
G1 × G2	6	0.72	2284.0	4.4	− 3.1	32.8	− 21.0	− 25.3
G1 × G6	6	0.71	2279.3	19.7	4.2	32.5	− 21.2	46.3
G2 × G4	4	0.73	2266.0	− 1.7	− 8.5	31.8	− 21.6	− 4.9
G1 × G3	3	0.78	2257.9	17.1	8.9	31.3	− 21.9	247.8
G4 × G7	6	0.77	2235.3	− 1.9	− 8.4	30.0	− 22.7	− 74.4
G2 × G7	6	0.80	2227.7	− 1.6	− 5.3	29.5	− 22.9	13.8
G1 × G7	9	0.76	2221.0	3.2	− 4.1	29.2	− 23.2	75.2
G5 × G6	2	0.79	2151.1	9.8	− 4.1	25.1	− 25.6	107.8
G2 × G3	2	0.79	2017.4	− 1.5	− 12.4	17.3	− 30.2	− 59.0
G3 × G4	2	0.73	2014.3	− 2.1	− 12.5	17.1	− 30.3	− 18.4
G3 × G5	1	0.85	1966.8	− 1.3	− 10.3	14.4	− 32.0	− 61.0
G3 × G6	2	0.81	1921.3	8.7	− 4.6	11.7	− 33.5	− 18.8
G3 × G7	3	0.78	1835.5	− 8.5	− 17.1	6.7	− 36.5	− 156.1
Mean		0.75	2212.9	5.9	− 3.3	28.7	− 23.4	19.7

*HGY* Hybrid grain yield, *PMPH* Panmictic mid-parent heterosis, *PBPH* Panmictic better parent heterosis, *PCH* Panmictic commercial heterosis, *SCA* Specific combining ability.

### Magnitude of heterosis

The estimates of PMPH and PBPH for grain yield are presented in Table 7. Across the 91 population hybrids, PMPH ranged from − 21.7 to 62.1% with a mean of 6.4%, while PBPH ranged from − 32.5 to 43.0% with a mean of − 2.9%. Across 21 marker-based group crosses, PMPH ranged from − 8.5% (G3 × G7) to 20.2% (G2 × G6) with a mean of 5.9%, while for PBPH it was − 17.1% (G3 × G7) to 8.9% (G1 × G3) with a mean of − 3.3% (Table 6). Grain yield of the popular check hybrid ProAgro 9444 was 2891 kg ha^−1^ and one population hybrid (GB 8735 × ICMP 87307) had positive grain yield advantage (3.5%) over the check hybrid (Table 8).

**Table 7:** Panmictic better parent heterosis (above diagonal) and panmictic mid-parent heterosis (below diagonal) of 91 pearl millet population hybrids across three locations.

Populations	EC C6	ICMP 98107	NWC C2	ICMP 87237	GB 8735	GICKV 98771	ICMS 7704	ICMV 155	SOSAT C88	Sudan I	Ugandi	MRC G.blk	HHVBC	ICMP 87307
EC C6		− 32.51*	− 13.01	− 5.67	− 27.48	− 10.31	8.11	25.10	2.71	− 19.36	− 18.84	− 5.57	20.45	− 17.29
ICMP 98107	− 21.70		− 17.15	− 2.69	− 21.24	− 14.78	− 11.24	− 0.74	− 7.63	− 3.97	− 18.63	4.80	-17.52	− 6.49
NWC C2	− 7.87	− 8.70		16.97	− 10.44	6.67	8.61	− 1.61	− 15.02	− 21.50	− 10.06	− 0.55	1.44	− 2.96
ICMP 87237	4.11	2.90	22.21		11.47	9.28	7.43	9.87	11.17	− 16.53	− 2.65	− 12.43	2.87	6.78
GB 8735	− 18.80	− 17.98	− 4.99	13.27		7.09	− 6.10	− 3.00	11.90	− 7.69	− 5.28	− 13.80	8.91	29.86*
GICKV 98771	− 1.28	− 9.62	11.12	9.62	9.14		3.96	3.66	6.47	− 10.96	− 4.64	5.32	5.08	− 12.21
ICMS 7704	24.43	15.72	31.23*	34.37*	18.85	29.72*		42.99*	0.80	− 15.37	− 7.83	10.87	8.98	0.59
ICMV 155	27.34*	16.89	5.97	23.20	10.31	15.92	62.08**		− 1.95	− 5.05	− 19.51	24.99	9.63	− 14.06
SOSAT C88	15.89	− 4.63	− 9.10	13.95	12.90	9.45	28.43*	12.35		− 17.72	− 18.52	5.37	1.07	2.48
Sudan I	− 3.54	− 0.40	− 10.61	− 8.65	− 0.44	− 2.28	13.17	15.20	− 11.99		− 1.54	− 20.66	− 16.43	− 6.73
Ugandi	− 5.54	− 18.33	− 0.55	3.30	− 1.01	1.48	20.48	− 4.93	− 15.57	1.76		− 17.75	− 12.19	− 17.58
MRC G.blk	− 0.64	16.22	0.15	− 7.90	− 7.96	10.45	33.22*	33.74**	13.46	− 9.12	− 8.50		1.90	− 3.10
HHVBC	29.48*	− 10.41	3.05	5.85	13.82	7.80	33.33*	19.80	6.52	− 6.15	− 4.30	4.23		12.87
ICMP 87307	− 6.95	− 3.14	3.49	9.10	30.59**	− 10.04	27.84*	− 1.80	2.83	0.07	− 14.32	4.00	18.58	

*, **Significant at 0.05 and 0.01 levels of probability, respectively.

**Table 8:** Panmictic commercial heterosis (%) over hybrid check ProAgro 9444 (above diagonal) and OPV check ICTP 8203 (below diagonal) of 91 pearl millet population hybrids across three locations.

Populations	EC C6	ICMP 98107	NWC C2	ICMP 87237	GB 8735	GICKV 98771	ICMS 7704	ICMV 155	SOSAT C88	Sudan I	Ugandi	MRC G.blk	HHVBC	ICMP 87307
EC C6		− 42.21**	− 39.30**	− 28.00**	− 42.83**	− 31.96**	− 32.99**	− 22.45*	− 17.58	− 25.61**	− 29.98**	− 35.02**	− 13.24	− 34.08**
ICMP 98107	− 2.85		− 29.05**	− 16.67	− 32.55**	− 27.02**	− 24.00*	− 15.00	− 20.90*	− 11.42	− 29.80**	− 10.26	− 29.37**	− 19.93*
NWC C2	2.04	19.26		− 10.72	− 29.40**	− 19.08*	− 24.21*	− 31.34**	− 31.81**	− 27.59**	− 22.41*	− 30.60**	− 26.93**	− 22.65*
ICMP 87237	21.03	40.07*	50.08**		− 12.13	− 16.59	− 18.00	− 16.14	− 10.80	− 23.00*	− 16.02	− 33.16**	− 21.48*	− 14.89
GB 8735	− 3.91	13.37	18.67	47.70**		− 15.59	− 25.99**	− 23.54*	− 10.21	− 14.84	− 18.29	− 32.06**	− 14.15	3.51
GICKV 98771	14.37	22.67	36.02*	40.21*	41.89**		− 21.14*	− 21.36*	− 14.57	− 17.87	− 17.74	− 20.11*	− 20.28*	− 30.03**
ICMS 7704	12.65	27.76	27.40	37.83*	24.41	32.56*		− 14.48	− 19.11*	− 21.93*	− 20.49*	− 23.71*	− 21.50*	− 19.83*
ICMV 155	30.36	42.88**	15.41	40.96*	28.53	32.18*	43.76**		− 21.33*	− 12.41	− 30.56**	− 13.99	− 21.03*	− 31.50**
SOSAT C88	38.54*	32.96*	14.62	49.95**	50.94**	43.61**	35.97*	32.25*		− 24.10*	− 29.71**	− 15.45	− 18.90*	− 17.76
Sudan I	25.04	48.91**	21.73	29.43	43.15**	38.06*	31.23	47.23**	27.59		− 9.17	− 26.82**	− 22.91*	− 13.96
Ugandi	17.70	18.00	30.43	41.17*	37.35*	38.28*	33.66*	16.72	18.15	52.68**		− 29.05**	− 24.25*	− 28.90**
MRC G.blk	9.23	50.86**	16.66	12.35	14.21	34.30*	28.24	44.58**	42.13**	23.02	19.27		− 26.60**	− 22.77*
HHVBC	45.84**	18.72	22.83	31.99*	44.31**	34.00*	31.96*	32.75*	36.33*	29.59	27.33	23.38		− 10.03
ICMP 87307	10.81	34.59*	30.02	43.07**	74.00**	17.62	34.77*	15.15	38.23*	44.63**	19.51	29.82	51.23**	

*, ** Significant at 0.05 and 0.01 levels of probability, respectively.

The range of panmictic commercial heterosis (PCH) in ProAgro 9444 ranged from − 42.83% (EC C6 × GB 8735) to 3.51% (GB 8735 × ICMP 87237) (Table 8), while in group crosses it ranged from − 14.8% (G2 × G6) to − 36.5% (G3 × G7) (Table 6). PCH in the popular OPV check (ICTP 8203, grain yield of 1720 kg ha^−1^) varied from − 3.9% (EC C6 × GB 8735) to 74.0% (GB 8735 × ICMP 87237) at individual hybrid level; at group level, G2 × G6 had the highest grain yield advantage of 43.2% followed by G2 × G5 (42.8%) and G4 × G5 (36.2%) (Table 6). Of the 91 population hybrids, 46 hybrids had significantly positive heterosis over OPV check ICTP 8203, whereas only 1 hybrid had positive heterosis over hybrid check ProAgro 9444. There was significant negative but low correlation (*r* = − 0.34, *P* < 0.001) between grain yield of population hybrids and GD between parental populations (Table 9). However, low positive correlation was found between grain yield of population hybrids and mean grain yield of parental lines (*r* = 0.20, *P* < 0.06) (Table 9).

**Table 9:** Correlation between genetic distance (GD), hybrid performance, combining ability effects and heterosis for grain yield.

Parameters	GD	HGY	MPGY	BPGY	GCA	SCA	PMPH
HGY	− 0.19	–					
MPGY	0.24*	0.20	–				
BPGY	0.07	0.11	0.78***	–			
GCA	− 0.09	0.53***	0.70***	0.57***	–		
SCA	− 0.17	0.82***	− 0.24*	− 0.26	− 0.05	–	
PMPH	− 0.34**	0.58***	− 0.68***	− 0.56***	− 0.17	0.79***	–
PBPH	− 0.21*	0.69***	− 0.43***	− 0.64***	− 0.02	0.82***	0.87***

*GD* Genetic distance, *HGY* Hybrid grain yield, *MPGY* Mid-parent grain yield, *SCA* Specific combining ability, *PMPH* Panmictic mid-parent heterosis, *PBPH* Panmictic better parent heterosis.

*, **, ***Significant at 0.05, 0.01, < 0.001 levels of probability, respectively.

### Heterotic pool formation

Hybrids of the G2 × G6 marker group crosses had the highest mean grain yield (2462 kg ha^−1^), PBPH (6.8%) and PMPH (20.2%) followed by marker group crosses of G2 × G5, G4 × G5, G1 × G4, G1 × G5 and G4 × G6 (Table 6). The lowest yielding hybrids were from G3 × G7 (1836 kg ha^−1^, − 17.1% PBPH, − 8.5% PMPH, − 156.1 SCA and − 116.7 GCA). Majority of marker group crosses involving the G3 group had below average grain yields, low heterosis and negative combining ability estimates (Table 6).

Pearl millet populations from the G2 group showed higher levels of heterotic parameters (hybrid yield performance*,* PMPH, PBPH, mean GCA and SCA) when crossed with populations of the G6 group (G2 × G6) followed by with the G5 group (G2 × G5). In addition, the G2 group when crossed with the populations of the other six groups, had highest mean grain yield of 2301 kg ha^−1^ followed by G5 and G6. Group G3 produced hybrids with lowest mean grain yield (Table 10).

**Table 10:** Mean grain yield of all the population hybrids when the representative parental population of each marker group were crossed to the rest of the marker groups, along with PMPH, PBPH and PCH in pearl millet.

Marker Group	HGY (kg ha^−1^)	PMPH (%)	PBPH (%)	PCH over ICTP 8203 (%)	PCH over ProAgro 9444 (%)	SCA
G2	2301	4.8	− 2.3	33.8	− 20.4	28.7
G5	2283	6.3	− 0.4	32.8	− 21.0	83.8
G6	2277	16.2	0.9	32.4	− 21.2	20.3
G1	2267	8.4	− 1.0	31.8	− 21.6	21.2
G4	2261	2.7	− 6.5	31.5	− 21.8	− 25.6
G7	2221	2.8	− 4.7	29.1	− 23.2	− 4.0
G3	2012	2.7	− 7.2	17.0	− 30.4	1.6

*HGY*, Hybrid grain yield, *PMPH* Panmictic mid-parent heterosis, *PBPH* Panmictic better parent heterosis, *PCH* Panmictic commercial heterosis, *SCA* Specific combining ability.

## Discussion

The high mean values observed for number of alleles detected, H_e_, H_o_ and PIC in this study indicated that the populations involved were quite diverse. The overall grouping pattern of 45 African and Asian populations showed that most of the Af-As populations formed distinct groups while most of the As-As and Af-Af populations were found in multiple marker-based groups and were found intermixed in the common groups^46^. Such a lack of clear-cut grouping based on geographical origin has also been reported in earlier studies based on molecular and phenotypic data among pearl millet populations of African^8–10,17^ and Asian regions^11,14,47^. Most of these studies indicated genetic admixture to be the main reason for such mixed grouping. The lack of differentiation among Asian or African populations was probably due to the high outcrossing nature of pearl millet leading to the concomitant high rate of pollen-mediated gene flow within the regions. The presence of these regional (As-As or Af-Af) populations in common cluster may be due to the high frequency of seed exchange of landraces across the regions in the past. The high out-crossing rate, as indicated by the high level of heterozygosity, increases the admixture level within the regions. Previous genetic diversity studies in pearl millet have shown high within population diversity, most likely caused by pollen-mediated gene flow and/or by seed-mediated gene flow^14,15^.

Analysis of variance for grain yield revealed highly significant variance due to environments (locations) indicating that the materials were evaluated under diverse environments. Analysis of variance for 91 population hybrids and their 14 parental populations revealed that the genotypic variation due to hybrids, parents and hybrid vs parents were highly significant for grain yield, indicating the prevalence of adequate genetic variation in pearl millet parental populations and population hybrids for grain yield. Significant hybrids vs parents variance indicated the presence of significant heterosis for grain yield in the population hybrids. Environment × hybrid and environment × parent interactions were highly significant for grain yield, indicating it was highly influenced by the environment^46^ (data for analysis of variance (ANOVA) was taken from a study by Patil et al.^46^).

The σ^2^_*GCA*_/σ^2^_*SCA*_ variance ratio (0.39) and predictability ratio (0.44) for grain yield indicated that it was largely controlled by dominance effects (Table 4). The significant values of both GCA and SCA effects indicated the presence of both additive and non-additive gene effects, but higher magnitude of SCA variance for grain yield demonstrated the relative predominance of non-additive gene effects. Earlier studies in pearl millet have reported predominantly non-additive genetic control for grain yield due to low GCA/SCA variance ratio^48,49^. Pucher et al.^24^ and Sattler et al.^25^ have reported higher magnitude of SCA variance for grain yield in West African pearl millet populations and their hybrids. On the contrary, Ouendeba et al.^23^ reported higher magnitude of GCA variance than SCA variance for grain yield among African pearl millet landraces. In our study, the populations under investigation had diverse genetic backgrounds and were geographically distant (Asian/African regions), while Ouendeba et al.^23^ studied only five improved populations belonging to a specific African geographical region. This might be the reason for the differences in the GCA/SCA variance ratio in these two studies, as several studies in other crops have also shown higher SCA than GCA among hybrids derived from multiple, divergent and geographically distinct populations compared to hybrids produced using parents from geographically related and/or highly recombined germplasm^50–52^.

Low GCA/SCA variance ratio also indicated that prediction accuracy of hybrid performance based on GCA would be less reliable and cannot support early testing and selection of parental populations based on the progeny’s GCA. To overcome this problem, Pucher et al.^24^ and Sattler et al.^25^ recommended a two-step selection procedure in West African pearl millet population hybrids—first select potential hybrid parents based on GCA and then evaluate crosses among the best combiners from the opposite heterotic pools, to identify the best performing hybrids based on both GCA and SCA effects. This process could be a way to increase prediction accuracy using GCA in the long run since the prediction accuracy of hybrid performance based on GCA effects is more accurate^53^. Heterotic groups once established will also increase the σ^2^_*GCA*_/σ^2^_*SCA*_ ratio as reported in previous studies in single cross hybrids^54–56^ and population hybrids^57^ belonging to opposite heterotic groups in maize. Melchinger and Gumber^54^ state that the formation of initial heterotic groups based on combining ability patterns developed from the representative populations selected based on diversity of large number of populations will help in developing a sustainable hybrid breeding program through the exploitation of heterosis in the diverse populations.

PMPH for grain yield had significant variation from − 21.7 to 62.1% with a mean of 6.4% across the 91 population hybrids. Population hybrid GB 8735 × ICMP 87307 had a grain yield advantage of 3.51% over the best hybrid check (Proagro9444) and 74% over the best OPV check (ICTP 8203) across all the locations. Two combinations (GB 8735 × ICMP 87307 and ICMS 7704 × ICMV 155) showed significant positive PBPH of 29.9% and 43.0%, respectively. These population hybrids can be used for recurrent selection to improve combining ability effects and can further be used to develop OPVs in regions where they are being cultivated. They can also be used as base parental populations to derive superior inbreds for a hybrid breeding program in pearl millet.

The lower mean PMPH of 6.4% for grain yield was found comparable to the results reported by Presterl and Weltzien^27^ with low mean PMPH of 2.41% among intercross population hybrids of Indian and African origin landraces/populations. Bidinger et al.^58^ also observed lower range of panmictic heterosis (− 11 to 17%) for grain yield among the top cross hybrids involving seed parents (A-lines) and Indian landrace pollinators. Low mean PMPH for grain yield was also reported in maize population hybrids by Silva and Miranda Filho^59^ who explained it might be because panmictic populations (pools, synthetics and composites) had most of the loci controlling different traits with intermediate allele frequencies with lower proportion of fixed alleles, thus leading to low expression of heterosis. Since heterosis is a function of the difference between allele frequencies, most loci contribute little to heterosis expression even under dominant gene action^60^. Moreover, the parental populations in our study had originated from very diverse agro-ecologies of Asia and Africa, resulting in their diverse plant architecture and adaptive ability to different geographies, which can lead to coadapted gene complexes at many loci behaving in epistatic manner leading to low heterosis values, as suggested by Presterl and Weltzein^27^. The other reason for low heterosis explained by Presterl and Weltzein^27^ is the cancellation of heterosis effects due to bidirectional dominance, leading to the balancing out of positive and negative heterosis among population hybrids, which is very common when dealing with a quantitative trait such as grain yield.

On the contrary, recent studies on population hybrids produced by intercrossing West African landrace populations reported high mean PMPH for grain yield^24,25,61^. Yadav^29^ too observed higher mean heterosis of 17% for grain yield among hybrids produced by crossing elite populations and landraces. These studies broadly indicate the general superiority of pearl millet hybrids over parental populations. In our study, though mean PMPH was low, about one-fourth of the hybrids (25 of 91) had > 15% PMPH. Hence, careful selection among these combinations of parental populations with high PMPH can lead to the development of productive cultivars. Such crosses are potential genetic material to broaden the germplasm base of pearl millet and to develop material for diverse adaptation.

A negative significant correlation (*r* =  − 0.34, *P* < 0.001) was observed between SSR-based GD of all the 91 population hybrids and PMPH (Table 9). In the case of pearl millet hybrid parents, Chowdari et al.^47^ and Gupta et al.^62^ did not find significant correlation between marker-based genetic distances and mid-parent yield heterosis based on Randomly Amplified Polymorphic DNA (RAPD) and SSR marker systems, respectively. Also, in the case of West African pearl millet populations, correlation between SSR-based Modified Roger’s Distance (MRD) and PMPH was found to be non-significant^25^. These studies indicated that GD-based prediction for grain yield heterosis was not possible in pearl millet. On the contrary, a significant positive association was reported between genetic distance and yield heterosis in hybrid parents of pearl millet^40^ and also in maize populations^42,43^. In the present study, the lack of association between GD and heterosis might be due to the use of a set of neutral markers since non-neutral markers linked to yield related QTLs could find a relationship between GD and PMPH more accurately^63,64^.

An important result of this study was the low but positive correlation between grain yield of hybrids and mean grain yield of parental populations, indicating that continuous selection for high yielding parental populations should be pursued in pearl millet breeding programs in order to boost the development of high yielding cultivars. Also, the mean grain yield of parental populations was found positively correlated with GCA for grain yield (*r* = 0.70, *P* < 0.001), indicating that selection of parental populations with high grain yield will lead to indirect selection for high GCA. A similar kind of association ws found by Gupta et al.^41^ in hybrid parental lines of pearl millet.

Parental populations of the G2 group, when crossed with parental populations of G5 and G6 groups, had highest hybrid performance, PMPH and PCH in comparison to other groups. In addition, the G2 group when crossed with the rest of the populations of all the six marker-based groups, had high hybrid yield performance followed by G5 and G6 groups. Hence, the G2 group was designated as PMPHP-1. As G5 and G6 groups showed similar hybrid yield performance, PMPH, PBPH and PCH as the G2 group, these two groups were merged and designated as PMPHP-2 (Fig. 1). PMPHP-1 represented marker group G2 which contained 6 populations (3 African and 3 Asian). The three African origin populations had a genetic background of West African germplasms, and the three Asian origin populations had a genetic background of ICRISAT-bred material crossed with Western Rajasthan germplasms. PMPHP-2 was found linked to G5 and G6 marker groups, each comprising 5 and 6 Af-As populations, respectively, which were developed at ICRISAT, Hyderabad using African germplasms with a genetic background of Smut Resistant Composites (SRC) and Bold Seeded Early Composites (BSEC) germplasms. This study could identify a heterotic pool pattern in pearl millet populations, while previous efforts of Pucher et al.^24^ and Sattler et al.^25^ failed to form heterotic pools in West African pearl millet populations. They reported high levels of genetic admixture in naturally occuring populations or released OPVs as the cause for their failure to identify heterotic pools, which was not the case in our study. The populations involved in this study were populations bred at ICRISAT which were maintained in isolation following proper guidelines.

The population hybrid combinations of two identified heterotic pools, PMPHP-1 × PMPHP-2, demonstrated higher mean hybrid performance (2458 kg ha^−1^) and PMPH (14.75%) compared to the mean of all hybrids’ performance (2213 kg ha^−1^) and overall heterosis of 5.9% for grain yield. These superior heterotic pool combinations had 2 Af-Af (SOSAT C88 and GB 8735), 3 As-As (Raj 171, CZP 86 and HiTiP 88) and 1 Af-As (ICMP 87703) populations in PMPHP-1 (G2), while PMPHP-2 (G5 and G6) had 10 Af-As and only 1 As-As populations. This indicated that crosses involving Af-As populations with either Af-Af or As-As populations displayed superior hybrid performance than crosses involving parental populations from the same region (As-As or Af-Af). These marker group population crosses (G2 with G5 and G6) showed high mean performance, heterosis and positive GCA as well as SCA effects indicating that these are potential heterotic pools from which to derive superior heterotic inbred lines. The group crosses between populations of G2 with G5 and G6 also showed higher SCA values than GCA values; a similar result of high SCA variance compared to GCA was reported by Sattler et al.^25^ in West African pearl millet population hybrids.

Estimates of GCA and SCA have been used extensively in maize population improvement programs as recurrent selection methods were designed to provide systematic, incremental genetic improvement in genetically broad-based populations for complex traits^65^. For breeding programs emphasizing the development of inbred lines and hybrids from populations derived from distinct heterotic groups, Hallauer^65^ suggested the use of reciprocal recurrent selection methods to enhance the performance of heterotic pattern. Similarly, the pearl millet population heterotic pools PMPHP-1 × PMPHHP-2 can also be subjected to reciprocal recurrent selection in which the population from one heterotic pool will serve as a tester for the population from the other heterotic pool to improve the GCA and SCA of both the populations in different heterotic pools. Such an approach will lead to the development of inbred lines to deliver hybrids with yields higher than those currently available. In addition, these crossing patterns could be used to broaden the genetic base within the hybrid parental line development programs to develop superior hybrid parents with higher productivity.

We also attempted to assign the established heterotic pools to seed (B-) or pollinator parent (R-) gene pools for the development of specific hybrid parents based on their fertility restoration ability on CMS sources and other yield related traits. Populations of PMPHP-1 were bold seeded, high yielding and had good maintainer ability for A_1_ CMS system, and were hence proposed for use in B-line development. Populations of the opposite heterotic pool PMPHP-2 showed high fertility restoration with comparatively lesser yield and 1000-seed weight; so they were proposed for R-line development in the hybrid breeding program (data not provided).

## Conclusion

The results of this study suggest that molecular markers can be used to group pearl millet populations into genetically similar groups, but heterosis cannot be predicted based on GD. The study was able to identify distinct heterotic groups PMPHP-1 and PMPHP-2 in pearl millet populations; pearl millet breeding programs can use these unused superior populations/gene pools strategically to develop highly productive hybrid parents. High heterosis shown by the crosses involving African origin and Asian-bred populations (Af-As) indicated that the exchange/introduction of African/Asian germplasm should be continued in breeding programs to enhance genetic gains in the future. The populations identified from opposite heterotic pools should be subjected to population improvement methods like reciprocal recurrent selection for a few cycles to help derive superior heterotic inbred lines.

## Material and methods

### Plant material

A large number of OPVs and several trait-based composites (e.g. early composite, medium composite, late composite, smut-resistant composite, high-tillering composite, bold-seeded composite, dwarf composite and high head volume composite) were developed by ICRISAT using a diverse range of germplasm from Asian and African sources through recurrent selection. A set of 45 diverse pearl millet populations were evaluated in this study (Supplementary Table 1). The methodology and the breeding materials involved are available in a recently published study by Patil et al.^46^.

### DNA extraction and SSR genotyping

Genomic DNA was isolated from leaf tissue of 16 randomly selected individuals from each population. A set of 720 DNA samples (45 populations × 16 individuals) were isolated along with the control sample Tift 23D_2_B_1_ using NucleoSpin® 96 Plant II Kit (Macherey-Nagel, Germany). Electrophoresis (0.8% agarose gel) was performed to test the quality of the DNA and quantified based on lambda DNA (MBI Fermentas, USA). The final working DNA samples were normalized uniformly at a concentration of 10 ng/µl.

Twenty-nine SSR markers (Supplementary Table 2), identified as highly polymorphic and distributed over all the seven linkage groups based on earlier studies^66,67^ were used. The detailed methodology followed for DNA extraction, SSR genotyping protocol and allele calling using Genemapper 4.0 (Applied Biosysterms) is explained in Patil et al.^46^.

### Parent selection and hybrid development

Genetic distance was estimated based on the MRD and a cluster diagram was developed for all the 45 populations using DARwin-5.0 software^68^. Clustering pattern delineated all the 45 populations into seven groups (designated as G1 to G7 marker groups) (Fig. 1). The pairwise *Fst* method was used to infer the distinctness of the groups in a neighbor-joining tree. Details of the methodology followed for the selection of representative parental populations from seven marker groups is explained in Patil et al.^46^.

The 14 representative populations (representing G1 to G7 marker groups) were crossed in full diallel mating design at ICRISAT during the 2016 summer season. All the possible 182 population hybrids were made by crossing parental populations in both the directions. At least 15 to 20 panicles from each parent were pollinated with bulk pollen collected from 20 to 25 plants of the respective crossing parent to avoid genotypic sampling effects in the parental populations. To generate enough seeds for multilocation evaluation, an equal quantity of seeds of each direct cross and its reciprocal cross were bulked together to develop 91 population hybrids. Based on the seven marker groups crossed in diallel fashion, these 91 population hybrids represented 21 marker-based group crosses.

### Experiment layout

A trial comprising 91 population hybrids, 14 parental populations, 4 standard hybrid checks (Kaveri Super Boss, Pioneer 86M86, ProAgro 9444 and ICMH 356) and 2 OPV checks (ICTP 8203 and Dhanashakti) was evaluated in an alpha lattice design with two replications during the rainy season (June to October) of 2016 at three locations in India. The locations were ICRISAT, Hyderabad, Telangana (17° 30′ N, 78° 27′ E, 545 m altitude), Regional Agricultural Research Station, Palem, Telangana (16° 53′ N, 78° 23′ E, 545 m altitude) and Pearl Millet Research Station, Junagadh Agricultural University, Jamnagar, Gujarat (22° 28′ N, 70° 04′ E and 27.6 m altitude). The population hybrids and parental populations were evaluated in separate blocks planted side by side in a replication to avoid the suppressive effect of hybrids on parental populations due to the vigorous growth of hybrids. Each entry was planted in 4 rows of 4 m length, with an inter-row spacing of 75 cm and 15 cm between plants. All the recommended agronomic practices for good crop growth were followed at all the locations. All the panicles in a plot were harvested for each entry separately. The harvested material was sundried for 10 to 15 days, threshed and recorded for grain yield in kilogram per plot (kg plot^−1^) and converted to grain yield in kilogram per hectare (kg ha^−1^). This experimental layout has been mentioned in Patil et al.^46^.

### Statistical analysis

#### Phenotypic analysis

Combined ANOVA was carried out using PROC MIXED^69^ with restricted maximum likelihood (REML) procedure, considering locations, genotypes and replications as fixed effects and blocks as a random effect (data for ANOVA was from a study by Patil et al.^46^). In order to combine the data across locations, individual location variances were modeled to error distribution using repeated statement in SAS mixed procedure. Variance components were estimated for GCA, SCA and their interactions with the environment (GCA × Environment and SCA × Environment) from multi-environment diallel method-II (half diallel with parents)^70^ using SAS PROC MIXED procedure^68^. Since parents in this study are panmictic populations, mid-parent heterosis was calculated as PMPH^71^. Furthermore, PBPH and PCH, analogous to better parent and commercial heterosis, were used. Genetic distance, SCA and PMPH between any of the 21 marker-based groups were also estimated based on the mean values of GD, SCA and PMPH, respectively, of all the probable combinations between representative parental populations in those two groups. Heterosis for grain yield was estimated as (i) PMPH = 100 × (F_1_ – MP)/MP; (ii) PBPH = 100 × (F_1_ – BP)/BP and (iii) PCH = 100 × (F_1_ – CC)/CC; where F_1_ is the hybrid yield, MP is the mean grain yield of both the parental populations, BP is the grain yield of the better yielding parental population and CC is the grain yield of the popular commercial check. ProAgro 9444 has been one of the most widely adapted and stable commercial hybrid cultivated over a large area for nearly the last two decades and ICTP 8203 is a widely grown OPV in India. Hence, these two were considered as commercial checks to compare heterosis levels with population hybrids. The correlation coefficient of MRD with hybrid performance per se, GCA and heterosis were estimated using SAS PROC CORR^69^. Predictability ratio was computed following Baker^72^ to estimate the relative importance of GCA in explaining hybrid performance as mentioned below:

$$\mathrm{Predictability ratio }(\mathrm{PR}) = \frac{{2\upsigma }_{\mathrm{GCA}}^{2}}{{(2\upsigma }_{\mathrm{GCA}}^{2}+{\upsigma }_{\mathrm{SCA}}^{2})}$$

where ${\upsigma }_{\mathrm{GCA}}^{2}$ and ${\upsigma }_{\mathrm{SCA}}^{2}$ were variances due to GCA and SCA, respectively; ${(2\upsigma }_{\mathrm{GCA}}^{2}+{\upsigma }_{\mathrm{SCA}}^{2})$ is the total genetic variance of single cross progenies (F_1_)^70^.

#### Molecular analysis

The MRD between two populations^73^ was calculated as:

$$\mathrm{MRD}=\sqrt{\frac{1}{2m}\sum_{i=1}^{m}\sum_{j=1}^{{a}_{i}}{\left({p}_{ij}-{q}_{ij}\right)}^{2}}$$

where *m* refers to the number of markers; *p*_*ij*_ and *q*_*ij*_ are the allele frequencies of j^th^ allele at the i^th^ marker in the two populations; *a*_*i*_ is the number of alleles at the ith marker.

Analysis of molecular variance (AMOVA)^74^ was performed to partition molecular genetic variance into components attributed to variance between and within populations. All the analyses were carried out using R program statistical software^75^.

### Ethics declarations

This study did not involve human participants or animals.

## Supplementary Information

Supplementary Tables.
