# Supplementary material for: Heterotic pools in African and Asian origin populations of pearl millet [Pennisetum glaucum (L.) R. Br.]
Source: Sci Rep. 2021 Jun 9;11:12197. doi: 10.1038/s41598-021-91568-7 (PMC8190140; doi:10.1038/s41598-021-91568-7)
Supplement: Supplementary file 1 — Supplementary Tables. [file 41598_2021_91568_MOESM1_ESM.docx]

**Heterotic pools in African and Asian origin populations of pearl millet [*Pennisetum glaucum* (L.) R. Br.]**

K. Sudarshan Patil^1,2*^, K. D. Mungra^3^, Shashibhushan D.^2^, Anil Kumar Vemula^1^, Roma R. Das^1^, Abhishek Rathore^1^, S. K. Gupta^1^

*^1^International Crops Research Institute for the Semi-Arid Tropics (ICRISAT), Hyderabad, Telangana, India*

*^2^Professor Jayashankar Telangana State Agricultural University (PJTSAU), Hyderabad, Telangana, India*

*^3^Junagadh Agricultural University (JAU), Jamnagar, Gujarat, India*

**Corresponding author: sudarshan.gpb@gmail.com* +91 789 357 4901

**Supplementary Table 1.** Details of cluster-wise pedigree of 45 pearl millet populations

| **Pop. No.** | **Marker Group** | **Populations** | **Pedigree** | **Material involved/ origin** | **Bred at ecology** | **Developed by** |
| --- | --- | --- | --- | --- | --- | --- |
| P14 | G1 | AIMP 92901 | Bred by random mating 272 Bold Seeded Early Composite (BSEC) S_1_ progenies selected at Aurangabad and BSEC S_1_s bulk ex Pat/K91. | African | Asia | Bred by CT Hash and AG Bhasker Raj with state-program partners based at Aurangabad. Maharashtra |
| P36 | G1 | CZ-IC 9802 | Bred by random mating 14 early-maturing and high-tillering full-sib progenies of Early Raj Pop. developed from crosses involving early-maturing and high tillering materials. | Asian | Asia | Bred by E Weltzien and CAZRI-based partner |
| P41 | G1 | HHVBC | High Head Volume B-lines Composite developed by random mating 38 progenies derived from pedigree breeding in summer 1990. All these lines were of African origin | African | Asia | Bred by KN Rai and his support staff |
| P42 | G1 | ICMV 155 | ICMV 84400: Bred by random mating 59 plants of New Elite Composite (NELC) C4 selected at ICRISAT-Patancheru | African | Asia | Bred by Pheru Singh and his support staff' |
| P40 | G1 | ICTP 8203 | The open pollinated cultivar ICTP 8203 was developed from progenies selected from Togo population which was introduced in India from the ICRISAT-Burkina Faso Cooperative Programme in 1980 | African | Asia | Bred by KN Rai and his support staff; selected by Namibian famers for testing leading to the release of Okashana 1 |
| P43 | G1 | RCB-IC 948 | Bred by random mating 65 S_1_ progenies of WRajPop 88 x EC II | Asian | Asia | Originally bred by E Weltzien in collaboration with one or more Rajasthan-based partners |
| P35 | G1 | Ugandi | Developed by ICRISAT-Sudan from Serere Composite selections | African | Africa | Bred in Sudan by G Ejeta |
| P37 | G1 | WRajPop bmr | WRajPop brown mid-rib version | Asian | Asia | Bred by E Weltzien and CT Hash |
| P38 | G1 | WRajPop C2 | Developed at ICRISAT by random mating 29 Chadi type landrace entries selected from Western Rajasthan in 1988 | Asian | Asia | Bred by E Weltzien and CAZRI and RAU-Mandor-based partners |
| P15 | G2 | CZP 86 | Developed from crosses between ICRISAT-bred materials and germplasm from western Rajasthan | Asian + ICRISAT | Asia | ICRISAT and CAZRI |
| P44 | G2 | GB 8735 | Open pollinated variety developed by random mating four F_3_ lines derived from crosses involving Iniadi and Souna | African | Africa | Bred by K Anand Kumar at ISC |
| P16 | G2 | HiTiP 88 C1 | Developed at ICRISAT by random mating 13 high-tillering pollinators from ICRISAT pollinator collection and open pollinated variety (CZP 86). CZP 86 was developed from crosses between ICRISAT-bred materials and germplasm from western Rajasthan. | Asian + ICRISAT | Asia | Bred by JR Witcombe and E Weltzien, in collaboration with CAZRI breeder OP Yadav |
| P45 | G2 | ICMP 87703 | Developed in 1979 at ICRISAT-Patancheru by random mating 37 Smut-resistant lines derived from West African germplasm | African | Asia |  |
| P39 | G2 | Raj 171 | Bred by random mating 8 S_1_ progenies of IVC selected at ICRISAT-Patancheru | Asian | Asia | Originally bred by Pheru Singh and selected by the lead RAU millet breeder as one of the entries in AICMIP national trials based on its performance at several locations in Rajasthan in 1987 |
| P34 | G2 | SOSAT C88 | Open pollinated variety developed by random mating 248 S_1_ progenies from the composite Souna x Sanio | African | Africa | Bred by O Niangadou of IER-Mali and K Anand Kumar of ICRISAT-Niger, at ISC during the 1987-1988 post-rainy season |

**Supplementary Table 1. (cont.).**

| **Pop. No.** | **Marker Group** | **Populations** | **Pedigree** | **Material involved/ origin** | **Bred at ecology** | **Developed by** |
| --- | --- | --- | --- | --- | --- | --- |
| P2 | G3 | EC C6 | Developed in 1974 at ICRISAT-Patancheru by random mating 153 Indian entries and 41 exotic entries | Asian x African | Asia | Bred by D Andrews, SB Chavan, Pheru Singh, and/or KN Rai and their staff |
| P1 | G3 | ICMP 89130 | Developed in 1987 at ICRISAT-Patancheru by crossing between EC II, ICMV 87901, ICMV 87902 (both BSEC varieties) and ICMV 87119 (EC II variety) | African | Asia | Bred by JR Witcombe and his support staff at ICRISAT, India |
| P6 | G3 | ICMP 96132 | Large Grain Population | African, Asian & Other | Asia | Bred by E Weltzien and JR Witcombe |
| P22 | G3 | ICMV-IS 92222 | ICMV-IS 92222, an improved variety bred by KAK by reselection within a farmers local (Haini-Kirei) collected at Say, Niger. This improved version of Haini-Kirei was bred by S_1_ selection to eliminate shibras, select for better downy mildew resistance, and select for earliness. | African | Africa | Bred by K Anand Kumar at ICRISAT, Niger |
| P10 | G3 | SC1 C4 Bulk | Serere Composite 1 from Uganda | African | Africa | Bred in Uganda before ICRISAT was set up; subsequently subjected to several cycles of improvement at ICRISAT, India. |
| P13 | G3 | WC C3 Bulk | World Composite received from Kano Nigeria in 1975 | African | Africa | Bred by David Andrews |
| P28 | G4 | CZ-IC 618 | ICMV 95836 - Bred by random mating 13 S_3_s selected from ERajPop C2 at Mandor K94 for fertility restoration | Asian | Asia | Bred by E Weltzein and Mandor-based partners |
| P7 | G4 | ICMP 00552 | Developed in 1994 at ICRISAT-Patancheru from random mating 928 F_1_s and F_2_s from seven crosses (ICMV 155 x SenPop , ICMV 155 x ICMV 91059, SRC II C1 x SenPop, SRC II C1 x ICMV 91059, ERC II C0 x ICMV 91059, Lubasi C1 x SenPop, SenPop x ICMV 91059) | African | Asia | Bred by CT Hash and AG Bhasker Raj |
| P32 | G4 | ICMV 94132 | Bred by random mating 15 S_1_s of LaGraP C0 selected for Grain Yield across PHF/S93 and Gwa/K93 | African | Asia | Bred by CT Hash and AG Bhasker Raj with contributions from G Chauhan at the GICKV College of Agriculture, Gwalior, Madhya Pradesh |
| P21 | G4 | ICMV 94135 | Bred by random mating 12 S_1_s of LaGraP C0 selected for High Yield and Agronomic Score at PHF/S93. | African | Asia | Variety selected from initial cycle S1 progenies evaluated by E Weltzein group |
| P33 | G4 | MRC Gen.blk. | Derived by inter-mating high-tillering, early-maturity, and small-seeded inbred lines from ICRISAT and five State Agricultural Universities in Northern India | Asian | Asia | Bred by KN Rai in collaboration with IS Khairwal and AICPMIP-affiliated breeders in the five SAUs in northern India |
| P23 | G4 | Sudan I | Developed at ICRISAT-Patancheru by random mating F_1_s involving 3 parents (ICMV 91059, SenPop and Sudan Yellow) selected from the late population diallel trial in 1989/90 at Patancheru | African | Asia | Bred by CT Hash and AG Bhasker Raj |

**Supplementary Table 1. (cont.).**

| **Pop. No.** | **Marker Group** | **Populations** | **Pedigree** | **Material involved/ origin** | **Bred at ecology** | **Developed by** |
| --- | --- | --- | --- | --- | --- | --- |
| P26 | G5 | GICKV 96752 (JBV 3) | Bred by random mating best 15 Smut Resistant Composite II C3 full-sib progenies selected visually at ICRISAT-Patancheru in 1995 | African | Asia | Bred by CT Hash and AG Bhasker Raj in collaboration with G Chauhan at the GICKV College of Agriculture, Gwalior, Madhya Pradesh |
| P31 | G5 | GICKV 98771 | Bred by random mating 212 S_1_ progenies from C3 cycle of the Early Smut Resistant Composite II (ESRC II) selected for downy mildew resistance in greenhouse screening at ICRISAT-Patancheru and agronomic performance at Gwalior in 1996 | African | Asia | Bred by CT Hash and AG Bhasker Raj in collaboration with G Chauhan at the GICKV College of Agriculture, Gwalior, Madhya Pradesh |
| P18 | G5 | ICMV 221 | Bred by random mating 124 selected S_1_ progenies of Bold Seeded Early Composite (BSEC) from summer season drought trial | African | Asia | Bred by JR Witcombe and his support staff; tested as ICMV 88904 and MP 221 |
| P24 | G5 | Sudan II | Developed at ICRISAT-Patancheru by random mating F_1_s involving 8 parents (ICMV 91059, SenPop, ICMV 155, ICMP 91751, ICMV 155, AfPop 90, ICMP 87307 and Sudan Yellow) selected from the late population diallel trial in 1989/90 at ICRISAT-Patancheru | African | Asia | Bred by CT Hash and AG Bhasker Raj |
| P25 | G5 | WC-C75 | Bred from 7 full-sib progenies of World Composite selected at Coimbatore in 1975. Reselection at ICRISAT-Patancheru within a composite population originally developed in Nigeria. | African | Asia | Bred by DJ Andrews |
| P27 | G6 | CZ-IC 922 | ICMV 91122 - bred by random mating 14 EC C6 S_1_s selected at Jodhpur. | Asia | Asia | Bred by E Weltzein and RAU-Jodhpur-based partners |
| P29 | G6 | ICMP 87307 | Developed in 1974 at ICRISAT-Patancheru by random mating 79 visually selected superior crosses of mostly Indian x African origin | Asian x African | Asia |  |
| P30 | G6 | ICMP 96201 | Developed in 1994 at ICRISAT-Patancheru by crossing SRC II-C2, ESRC II-C2, IVC-C8 as females and ICMV 88908, ICMV 31293 as males | African | Asia | Population was initially generated by JR Witcombe and his support staff, and subsequently improved by CT Hash and AG Bhasker Raj |
| P17 | G6 | ICMS 7704 | Bred from 6 inbred lines derived from Indian x African crosses selected at Tandojan in Pakistan in 1977 | Asian x African | Asia | Bred by SB Chavan and his staff |
| P19 | G6 | ICMV 88908 | Mass selected variety bred from a cross of Bold Seeded Early Composite (BSEC) C4 and ICMV 87901, a variety from BSEC C3 | African, Asian & Other | Asia | Bred by JR Witcombe and his support staff; released as version of Okashana 1 in Namibia and elsewhere in Eastern and Southern Africa |
| P20 | G6 | ICMV 93752 | Bred at ICRISAT by random mating 16 S_1_ progenies from the C1 cycle of Smut Resistant Composite II (SRC II) selected for grain yield and agronomic score at ICRISAT-Patancheru in rainy season 1992 | African | Asia | Base population bred by JR Witcombe and support staff; variety developed by CT Hash and AG Bhasker Raj |
| P12 | G7 | ICMP 87237 | Developed at ICRISAT-Patancheru by random mating four composites [SC_1_(S_4_), SC_2_(M), SC_3_(M) and SC_4_(M)] which were developed at Serere Research Station, Uganda | African | Asia | Bred by JR Witombe and his support staff |
| P9 | G7 | ICMP 96601 | Developed in 1993 at ICRISAT-Patancheru from 702 S_1_ progenies from Zimbabwe. Selected 34 S_1_ progenies that were marked as long headed and sown for recombination by making full-sibs. | African | Asia | Originally bred by E Monyo, introduced to ICRISAT, India by E Weltzien before being passed on to the Population Improvement group |

**Supplementary Table 1. (cont.).**

| **Pop. No.** | **Marker group** | **Populations** | **Pedigree** | **Material involved/ origin** | **Bred at ecology** | **Developed by** |
| --- | --- | --- | --- | --- | --- | --- |
| P11 | G7 | ICMP 97754 | Developed in 1987 at ICRISAT-Patancheru by crossing IVC-C7 and SRC-C3 | African | Asia | Cycle 4 population bulk of SRC II, initially generated by JR Witcombe and later developed by CT Hash and AG Bhasker Raj |
| P4 | G7 | ICMP 97774 | Developed in 1989 at ICRISAT-Patancheru by random mating selected early progenies from SRC II at Hisar | African | Asia |  |
| P3 | G7 | ICMP 98107 | Developed in 1983 at ICRISAT-Patancheru by random mating 187 early-maturing progenies from 8 diverse composites [D1C, NEC, IVC, NELC, WC-C75, Togo, MC, SRC] and 17 open pollinated varieties | African | Asia | C7 population bulk; bred by Pheru Singh with input from D Andrews |
| P5 | G7 | ICMP 99901 | Developed in 1996 at ICRISAT-Patancheru from 110 half-sib progenies of HHVBC received from KN Rai | African | Asia | Bred by KN Rai |
| P8 | G7 | NWC C2 | Nigerian World Composite | African | Africa | Bred by D Andrews |

**Supplementary Table 2.** Details of multiplexing, primer sequence and linkage group of 29 SSR markers

| **Sl. No.** | **Multiplex** | **Markers** | **LG** | **Fluorescent dye** | **Repeat motif** | **Size range of the PCR products (bp)** | **Forward primer sequence (5'→3')** | **Reverse primer sequence (5'→3')** | **Reference** |
| --- | --- | --- | --- | --- | --- | --- | --- | --- | --- |
| 1 | 1 | *Xicmp3043* | 7 | 6-FAM | (AGC)5 | 198-228 | TCCTGTACAAGGACGTGCAG | TATCGACGCCAACGATACTG | Senthilvel *et al*., 2008 |
| 2 | 1 | *Xpsmp2079.2* | 7 | NED |  | 117-179 | CAGCCGAAGGCTAATCAACAA | GTGGTCAGCAGCAGATGTAA | Napoleon *et al*., 2012 |
| 3 | 1 | *Xpsmp2275* | 6 | PET | (GTT)10 | 267-300 | CCAGTGCCTGCATTCTTGGC | GCATCGAATACTTCATCTCA | Allouis *et al*., 2001 |
| 4 | 1 | *Xipes0082* | 7 | VIC | (AGGAG)7 | 154-209 | CGACCCCTGAAGGAAATCTT | TTCTTCATGTGGGTGTCGAA | Rajaram *et al*., 2013 |
| 5 | 2 | *Xipes0203* | 1 | 6-FAM | (ATC)16 | 229-307 | CCCTCGAAGAGATCGAAGTG | CTGAAACAACAGCCTGCAAA | Rajaram *et al*., 2013 |
| 6 | 2 | *Xpsmp2220* | 5 | NED | (GT)11 | 136-156 | GCATCCTTCACCATTCAAGACA | TGGGAAACAGAATGGAGAAAAGAG | Qi *et al*., 2001 |
| 7 | 2 | *Xpsmp2232* | 1 & 2 | PET | (TG)8 | 247-275 | TGTTGTTGGGAGAGGGTATGAG | CTCTCGCCATTCTTCAAGTTCA | Allouis *et al*., 2001 |
| 8 | 2 | *Xpsmp2070* | 3 | VIC | (CA)25 (TA)6 | 204-278 | AGAAAAAGAGAGGCACAGGAGA | GCCACTCGATGGAAATGTGAAA | Qi *et al*., 2004 |
| 9 | 3 | *Xicmp3088* | 1 | 6-FAM | (TCC)8 (TCTA)4 | 153-192 | TCAGGTGGAGATCGATGTTG | TTACGGGAGGATGAGGATG | Senthilvel *et al*., 2008 |
| 10 | 3 | *Xipes0220.1* | 3 | NED |  | 172-216 | CGTGGTCGATGGACTGCT | CGAGAGATTCACACCAAGCA | Rajaram *et al*., 2013 |
| 11 | 3 | *Xpsmp2207* | 7 | PET | (GT)5 | 315-333 | CAGGGCATACTTCAAGATTGATTC | GTCCACTTGTTATTCTCTATCACC | Qi *et al*., 2001 |
| 12 | 3 | *Xipes0186* | 4 | VIC | (TTG)10 | 169-293 | AGCATATGGCATCCTTTTCG | TTTCAGGCTTGGATTCAATGT | Rajaram *et al*., 2013 |
| 13 | 4 | *Xipes0152.2* | 5 | 6-FAM |  | 109-137 | TACGAAGGGAAGCACAGC | TGTGTGGTAAGCTGCTGGAG | Rajaram *et al*., 2013 |
| 14 | 4 | *Xpsmp2089* | 2 | PET | (AC)15 | 117-159 | TTCGCCGCTGCTACATACTT | TGTGCATGTTGCTGGTCATT | Qi *et al*., 2004 |
| 15 | 4 | *Xipes0200* | 6 | VIC | (GTAC)11 | 174-210 | GCGCTTTCAGAGTCCTGAGT | CAAGTCGTCACGGCCTTATT | Rajaram *et al*., 2013 |
| 16 | 5 | *Xpsmp2203* | 7 | 6-FAM | (GT)18 imp. | 351-389 | GAACTTGATGAGTGCCACTAGC | TTGTGTAGGGAGCAACCTTGAT | Qi *et al*., 2001 |
| 17 | 5 | *Xpsmp2085* | 4 | PET | (AC)11 | 181-199 | GCACATCATCTCTATAGTATGCAG | GCATCCGTCATCAGGAAATAA | Qi *et al*., 2004 |
| 18 | 5 | *Xpsmp2068* | 2 | VIC | (AC)14 | 112-162 | CAATAACCAAACAAGCAGGCAG | CTTCACTCCCACCCTTTCTAATTC | Qi *et al*., 2004 |
| 19 | 6 | *Xpsmp2201* | 2 | 6-FAM | (GT)6 | 363-385 | CCCGACGTTATGCGTTAAGTT | TCCATCCATCCATTAATCCACA | Qi *et al*., 2001 |
| 20 | 6 | *Xctm10* | 3 | PET | (CT)22 | 183-219 | GAGGCAAAAGTGGAAGACAG | TTGATTCCCGGTTCTATCGA | Budak *et al*., 2003 |
| 21 | 6 | *Xpsmp2202* | 5 | VIC | (GT)8 | 161-185 | CTGCCTGTTGAGAATAAATGAG | GTTCCGAATATAGAGCCCAAG | Qi *et al*., 2001 |
| 22 | 7 | *Xicmp3032* | 1 | 6-FAM | (GCT)8 | 202-217 | GCGTAGACGGCGTAGATGAT | CAACAGCATCAAGCAGGAGA | Senthilvel *et al*., 2008 |
| 23 | 7 | *Xipes0213* | 3 | VIC | (GAT)4 | 163-181 | GTCCGGTTTGTCTCTCCTTG | TGGATCTCCCATGTCGTGTA | Rajaram *et al*., 2013 |
| 24 | 8 | *Xipes0236* | 2 | 6-FAM | (TGG)11 | 215-263 | GGCCAGCTCGCCTAGAT | AGATCCACCGCCTAATGAAA | Rajaram *et al*., 2013 |
| 25 | 8 | *Xpsmp2249* | 3 | PET | (GT)7 imp. | 154-182 | CAGTCTCTAACAAACAAACACGGC | GACAGCAACCAACTCCAAACTCCA | Allouis *et al*., 2001 |
| 26 | 8 | *Xpsmp2086* | 4 | VIC | (AC)14 | 107-149 | CGCTTGTTTTCCTTTCTTGCTGTT | CCTTCTCAGATCCTGTGCTTTCTT | Qi *et al*., 2004 |
| 27 | 9 | *Xpsmp2030* | 1 | 6-FAM | (CA)11 (GA)10 | 102-160 | ACCAGAGCTTGGAAATCAGCAC | ATAATGCTTCAAATCTGCCACAC | Qi *et al*., 2004 |
| 28 | 9 | *Xpsmp2248* | 6 | PET | (TG)10 | 177-209 | TCTGTTTGTTTGGGTCAGGTCCTTC | CGAATACGTATGGAGAACTGCGCATC | Allouis *et al*., 2001 |
| 29 | 9 | *Xpsmp2090* | 1 | VIC | (CT)12) | 189-211 | AGCAGCCCAGTAATACCTCAGCTC | AGCCCTAGCGCACAACACAAACTC | Qi *et al*., 2004 |
| LG: linkage group on the pearl millet genetic map; imp: imperfect repeats; bp: base pairs | | | | | | | |  |  |
